# Establishment, optimisation and quantitation of a bioluminescent murine infection model of visceral leishmaniasis for systematic vaccine screening

**DOI:** 10.1038/s41598-020-61662-3

**Published:** 2020-03-13

**Authors:** Han Boon Ong, Simon Clare, Adam Jonathan Roberts, Mary Edythe Wilson, Gavin James Wright

**Affiliations:** 1 Cell Surface Signalling Laboratory, Wellcome Sanger Institute, Cambridge, UK; 2 Pathogen Laboratory Support, Wellcome Sanger Institute, Cambridge, UK; 3 Departments of Microbiology and Immunology and Internal Medicine, University of Iowa, and the Iowa City Veterans’ Affairs Medical Center, Iowa City, USA

**Keywords:** Parasitology, Vaccines, Infection, Parasitic infection

## Abstract

Visceral leishmaniasis is an infectious parasitic disease caused by the protozoan parasites *Leishmania donovani* and *Leishmania infantum*. The drugs currently used to treat visceral leishmaniasis suffer from toxicity and the emergence of parasite resistance, and so a better solution would be the development of an effective subunit vaccine; however, no approved vaccine currently exists. The comparative testing of a large number of vaccine candidates requires a quantitative and reproducible experimental murine infection model, but the parameters that influence infection pathology have not been systematically determined. To address this, we have established an infection model using a transgenic luciferase-expressing *L. donovani* parasite and longitudinally quantified the infections using *in vivo* bioluminescent imaging within individual mice. We examined the effects of varying the infection route, the site of adjuvant formulation administration, and standardised the parasite preparation and dose. We observed that the increase in parasite load within the liver during the first few weeks of infection was directly proportional to the parasite number in the initial inoculum. Finally, we show that immunity can be induced in pre-exposed animals that have resolved an initial infection. This murine infection model provides a platform for systematic subunit vaccine testing against visceral leishmaniasis.

## Introduction

Protozoa of the genus *Leishmania* are obligate intracellular parasites which cause the disease leishmaniasis. The parasite is transmitted by the bite of an infected female phlebotomine sand fly during a blood meal, and every year there are an estimated ~1 million new infections resulting in ~65,000 deaths^1^. Although the disease burden disproportionately affects poor people living in tropical and developing countries, the expanding geographic range of vector distribution caused by climate and environmental changes is an emerging threat outside these regions^2–4^. Outbreaks and re-emergence of leishmaniasis are also linked to other factors including political and socioeconomic upheavals^5–7^. Within the human host, *Leishmania* spp. cause a spectrum of species-specific clinical manifestations known as cutaneous, mucocutaneous or visceral leishmaniases, and it is the visceral form that is considered to be the most severe, and is ultimately fatal if left untreated. Symptomatic visceral leishmaniasis (VL), results from infections of *L. donovani* and *L. infantum*, and while representing only ~10% of all symptomatic leishmanial infections, they are responsible for nearly all leishmaniasis-attributed fatalities^1^. The current front-line drug treatments for VL: liposomal amphotericin B, miltefosine, paromomycin and antimonials, are far from ideal, with major concerns surrounding general toxicity or increased treatment failure^8,9^. While improved drugs are being developed^10,11^, the deployment of an effective vaccine would be an important disease control tool, but to date, no effective vaccine has been licensed for human leishmaniasis.

The earliest evidence of leishmaniasis being prevented by vaccination was the practice of leishmanisation where resistance to infections was induced by inoculating individuals with live parasites collected from the lesions of a cutaneous infection^12–15^. Patients who have recovered from self-healing primary infections or successful drug treatments often develop life-long immunity against re-infection^15,16^. The use of live organisms for vaccination, however, has significant safety concerns; consequently, modern vaccines are typically subunit vaccines: chemically defined products such as recombinant proteins that can elicit protective immune responses. Developing these subunit vaccines usually requires a suitable experimental model to systematically test candidates. While the disease progression of leishmania infections in dogs and hamsters is considered to more closely reflect the pathology of human VL infections than mice^17–19^, these animal models are difficult to use at the necessary scale because of ethical objections, lack of reagents and the difficulties with high costs of animal husbandry. To address these challenges, murine models for VL have been used, with infections using the susceptible BALB/c strain arguably being the best characterised^19–21^. Parasites primarily infect and multiply within liver macrophages of BALB/c mice during the first few weeks of infection, before an effective immune response is raised to clear the liver of parasites^22–24^. This is followed by a delayed but progressive increase in parasite burden within the spleen accompanied by splenomegaly; unlike the liver, however, spleen infections are persistent and indefinitely maintained. Genetically modified immuno-compromised mice such as *Rag1* and *Rag2*-deficient mice have also been used successfully as models for VL and unlike the BALB/c strain, these mice more closely reflect human disease pathology since they do not clear parasites from the liver^25–28^. Uses of these immunodeficient animals, however, are generally limited to studies involving drug trials or *in vivo* parasite propagation since they are incapable of generating adaptive immune responses.

Because of their genetic tractability and characterization, mice have become a useful model organism to characterise host factors and immune responses to *Leishmania spp*. infections. Despite these advantages, variations in infection pathology within a cohort of infected animals and the difficulty of repeatedly quantifying parasite loads in visceral organs within an individual animal are challenges to address for comparative vaccinology studies. Currently, the most commonly used techniques for parasite quantification are organ impression smears, limiting dilution^29^, and *q*PCR^30^ methods, but these methods require euthanizing the animal to access target organs limiting the amount of data that can be collected per individual. These experimental difficulties have been largely addressed by the use of *in vivo* bioluminescent imaging, where parasite burden and location can be longitudinally determined within individual mice by infecting animals with transgenic parasites that constitutively express a luciferase enzyme that generates light in the presence of the luciferin substrate. While several studies have used bioluminescent *Leishmania* spp. parasites, expressing different types of luciferase for a variety of purposes^31–35^, only a few have fully characterised the VL-infection progression *in vivo*^36–38^. Careful assessment and understanding of how different parameters such as parasite dose, route of administration or how the effects of adjuvant on vaccine formulation may affect experimental infections is important for these models to be useful for both drug and especially vaccine development.

Here, we establish a reproducible and highly quantitative murine infection model for evaluating vaccine candidates for VL by selecting a transgenic *L. donovani* parasite expressing the firefly luciferase and investigating the effects of experimental parameters that are known to influence the progression of infection, and which could affect comparative vaccine testing.

## Results

### The inoculation route of *L. donovani* infection influences disease pathology

**Figure 1:**
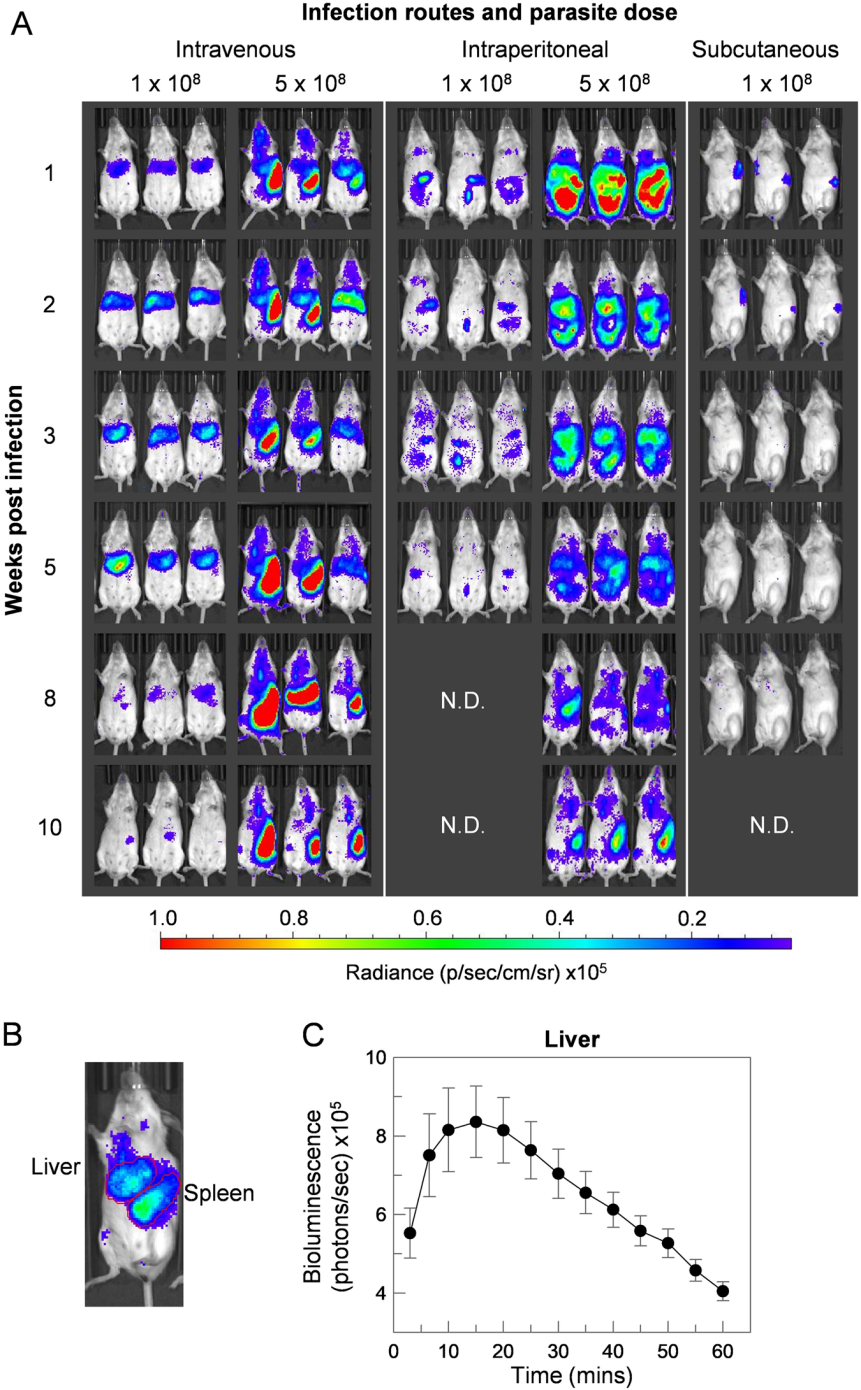
The route of parasite administration in experimental infections of *L. donovani* influenced disease pathology. (**A**) Groups of five mice were infected with either day five (intraperitoneal and intravenous delivery) or day seven (subcutaneous) stationary-phase transgenic luciferase-expressing *L. donovani* promastigote cultures at the indicated doses. Parasite loads were quantified at the indicated times by administering the luciferase substrate, luciferin, and the animals imaged using an IVIS instrument. Three representative animals from the group are shown and are arranged so that the infection dynamics can be followed in each individual animal throughout the course of the infection. The route of parasite administration and dose influenced the progression of the infection. **(B)** Parasite loads were separately quantified in the liver or spleen by defining these organs as shown and measuring the associated bioluminescence. **(C)** Liver bioluminescence increased over time following luciferin injection, peaked at between 10 to 20 minutes before a steady decrease in signal over the next hour. Data points represent means ± S.E.M. (*n* = 5, from individual mice).

To establish a reproducible and quantitative murine infection model of VL, we transfected the LV9 strain of *L. donovani* and selected a recombinant parasite that constitutively expressed the firefly luciferase gene to permit tracking and quantification of infected mice using *in vivo* imaging. As inoculation routes and doses are known to influence *L. infantum* infections of mice^39–42^, we first challenged groups of BALB/c mice with stationary phase promastigotes through intravenous, subcutaneous or intraperitoneal parasite delivery, and compared the resulting infections in each animal every week for ten weeks (Fig. 1A). Hepatic and splenic parasite loads were separately quantified by defining the regions on each animal corresponding to the liver and spleen (Fig. 1B), and the rate at which the luciferin substrate was oxidised to determine the optimum time for data acquisition after luciferin administration (Fig. 1C). By quantifying liver bioluminescence, total light flux increased steadily during the first 10 minutes, peaking between 10 to 20 minutes before a gradual decline. Intravenous delivery of parasites resulted in the parasitisation of the liver during the first few weeks of infection that resolved followed by dissemination and persistence within the spleen after the sixth week. We observed that a higher parasite dose resulted in the parasitisation of the spleen by week five, which was less evident in mice given a lower dose (Fig. 1A). To confirm that mice with bioluminescent signals were infected, we prepared impression smears from infected mouse spleens and were able to observe parasites within host cells. We noticed that it was difficult to find infected cells on the impression smears even from mice with high bioluminescence signals suggesting that bioluminescence was a much more sensitive method to quantify parasite loads (Fig. S1).

Immunocompromised *Rag1* and *Rag2*-deficient mice have been useful models to isolate and study components of host immunity to VL, as well as a source of *ex vivo* amastigotes^25–28^. To determine the virulence of transgenic *L. donovani* in immunodeficient *Rag1* knockout mice, they were infected intravenously with 10^8^ stationary phase promastigotes and the infection followed by *in vivo* imaging (Fig. S2A). By contrast to immunocompetent mice, *Rag1*-deficient mice were unable to resolve their liver infections resulting in a progressive increase in hepatic bioluminescence over the course of 14 weeks (Fig. S2B). *Rag1*-deficient mice lack mature lymphocytes and possess only a rudimentary spleen and so it is perhaps unsurprising that we observed little or no evidence of parasite-dissemination or persistence in the spleen. Indeed, splenomegaly was not observed in the post-mortem of these animals, suggesting parasite persistence requires adaptive an immune response.

To determine whether the route of parasite administration can affect the disease pathology in animals, parasites were injected either subcutaneously or into the peritoneal cavity. Mice infected subcutaneously showed little or no disease progression with observable parasites persisting at the site of inoculation before parasite loads fell below the IVIS detection limits after five weeks. Where parasites were inoculated into the peritoneal cavity at the lower dose, parasites remained within the cavity for several weeks with no obvious progression to the liver or spleen. At the high dose, parasites were detected in the liver and spleen (Fig. 1A); however, signals from parasites that persisted in the peritoneal cavity made localising and quantifying the parasite load in the liver and spleen difficult. We therefore concluded that intravenous delivery of *L. donovani* parasites resulted in the most reproducible infections that reflected what is known the disease pathology and progression in humans.

### Infections are dependent on the duration of parasite culture in stationary phase

**Figure 2:**
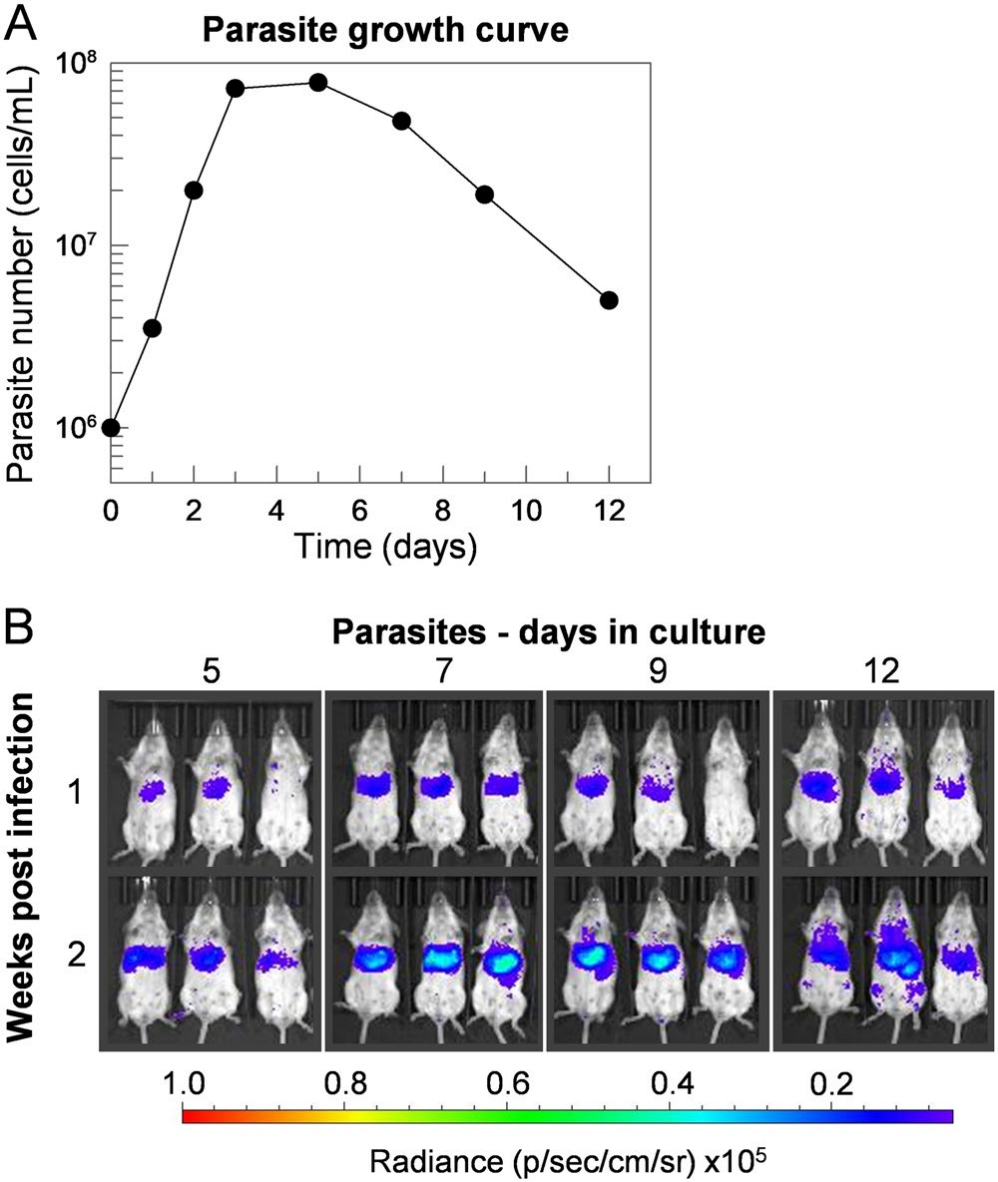
Infections are dependent on the duration of stationary phase parasite culture. (**A**) Fresh cultures of luciferase-expressing transgenic *L. donovani* parasites were established at an initial seeding density of 1 × 10^6^ cells/mL and growth was quantified over 12 days by counting live (motile) cells using a haemocytometer. After peaking at around days 3 to 7, motile parasite numbers declined. (**B**) Parasites were harvested at the specified time and used to infect groups of five mice intravenously. Infections were monitored during the initial 2–3 weeks by *in vivo* imaging. Three representative animals from the group are shown and are arranged so that the infection dynamics can be followed in each individual animal throughout the course of the infection.

The virulence of cultured *L. donovani* promastigotes in animals has previously been shown to be dependent on metacyclogenesis, a state that can be modelled during growth to stationary-phase^43^. To determine the optimal stationary phase time-point for infections, a growth analysis was performed on *L. donovani* promastigotes cultured in *Ld*Pro medium (Fig. 2A). Using an initial seeding density of 1 × 10^6^ cells/mL, parasites continued to grow exponentially for three days before the stationary phase was reached at approximately 8 × 10^7^ cells/mL. Stationary phase density was maintained for a further two days before the number of living cells, as determined by those that remained motile, declined steadily over the next seven days. To investigate the relationship between stationary-phase progression and parasite virulence, cells were harvested at regular time intervals (days 5, 7, 9 and 12) and used to infect BALB/c mice (Fig. 2B). Liver bioluminescence analyses of infected animals revealed that parasites harvested on days 7 and 9 of the culture were marginally more virulent compared to those harvested on days 5 and 12. Based upon these observations, parasites taken from stationary phase cultures between days 7 and 9 were considered optimal for infections, with day 7 cultures being used for all subsequent infection studies.

### The number of parasites in the inoculum affects disease progression and severity

**Figure 3:**
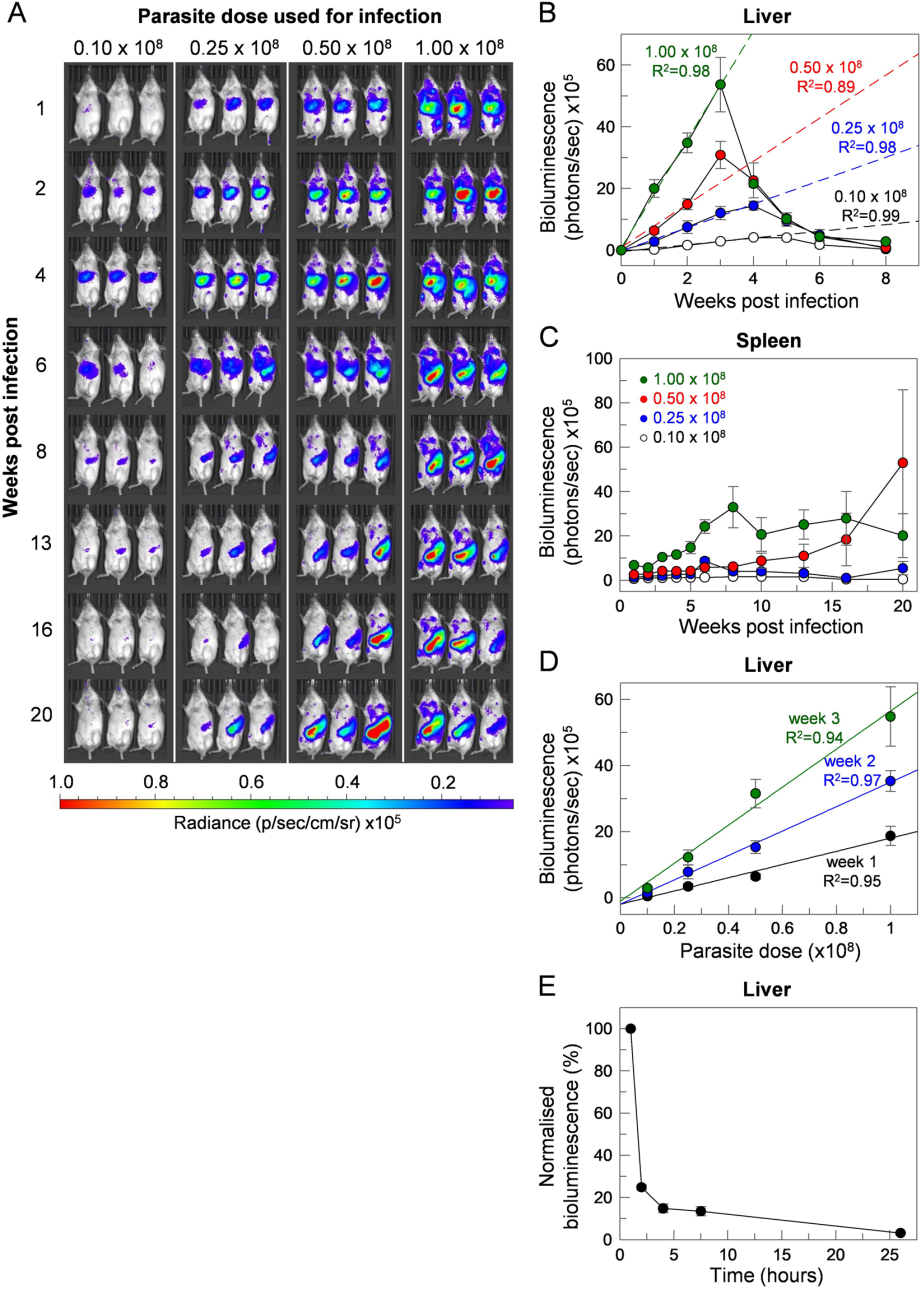
The progression and severity of *L. donovani* infection of mice is dependent upon the initial inoculum size. (**A**) Groups of three mice were infected intravenously with different doses of day-seven stationary-phase *L. donovani* promastigotes and infections were monitored over 20 weeks by *in vivo* imaging. To quantify the parasite load, the bioluminescence (total flux, photon/sec) associated with the liver (**B**) and spleen (**C**) was measured. Mice were infected with 0.10 × 10^8^ (white circles), 0.25 × 10^8^ (blue circles), 0.50 × 10^8^ (red circles) or 1.00 × 10^8^ (green circles) parasites, respectively. Liver-associated bioluminescence increased linearly during the initial 1–3 weeks, analysed using linear regression in GraFit and plotted as dashed lines. The progression of infection of the liver during the initial three weeks was also analysed using linear regression and increases in bioluminescence were found to be directly proportional to the parasite dose for weeks 1 (black circles), 2 (blue circles) and 3 (green circles), respectively (**D**). The parasites’ rapid localisation and clearance in the liver within first 24 h of infection were determined in mice challenged with 1 × 10^8^ parasites (*n* = 5) (**E**). Liver bioluminescence was normalised using measurements at 1 hour post infection as control for 100%. Data points represent means ± S.E.M. (*n* = 3 for B, C and D) or (*n* = 5 for E).

Given that our initial experiments had suggested the number of parasites in the initial inoculum affected subsequent disease progression, we extended these experiments and infected mice with a dose titration of parasites ranging from 0.1 to 1.0 × 10^8^ parasites per mouse (Fig. 3A). In general, parasite loads throughout the infection time course correlated with the initial dose (Fig. 3A), and was separately quantified in the liver (Fig. 3B) and spleen (Fig. 3C) using *in vivo* light-based imaging. We observed a linear increase in hepatic bioluminescence over the first three weeks of infection (Fig. 3B), which extended to the fourth week in mice infected with lower parasite doses, before resolving. The increases in liver bioluminescence were found to be directly proportional to the number of parasites used to initially infect the animals (Fig. 3D). Monitoring of mice infected with 1 × 10^8^ *L. donovani* parasites during the first 24 hours of infection revealed parasites to rapidly localise to the liver within the first hour, followed by a rapid decline in bioluminescence over the next two to eight hours (Fig. 3E). Of the initial signal in the liver one hour following infection, only a fraction (~3%) remained after 24 hours suggesting BALB/c macrophages are highly competent at parasite clearance. This observation may also be a result of only a fraction of the parasites being capable of successful differentiation from promastigotes to amastigotes to establish an infection in the mammalian host. Following this dramatic reduction, there was a linear increase in liver bioluminescence over the next three weeks which eventually resolved, falling below the limits of detection by between weeks six and eight. The reduction in hepatic parasite load was followed by dissemination to the spleen after week six, although signals in the spleen could be detected during the first six weeks in animals infected with the higher parasite doses. By contrast to the liver, parasitisation of the spleen between individual animals within the same group was more variable (Fig. 3C), although those animals receiving more parasites in the initial inoculum generally resulted in spleens with higher parasite loads. Given that 1 × 10^8^ parasites per mouse resulted in reproducibly robust infections of both liver and spleen, and was, in general, very well tolerated, this dose was used in all subsequent studies.

### The administration route of vaccine formulation affected disease progression

**Figure 4:**
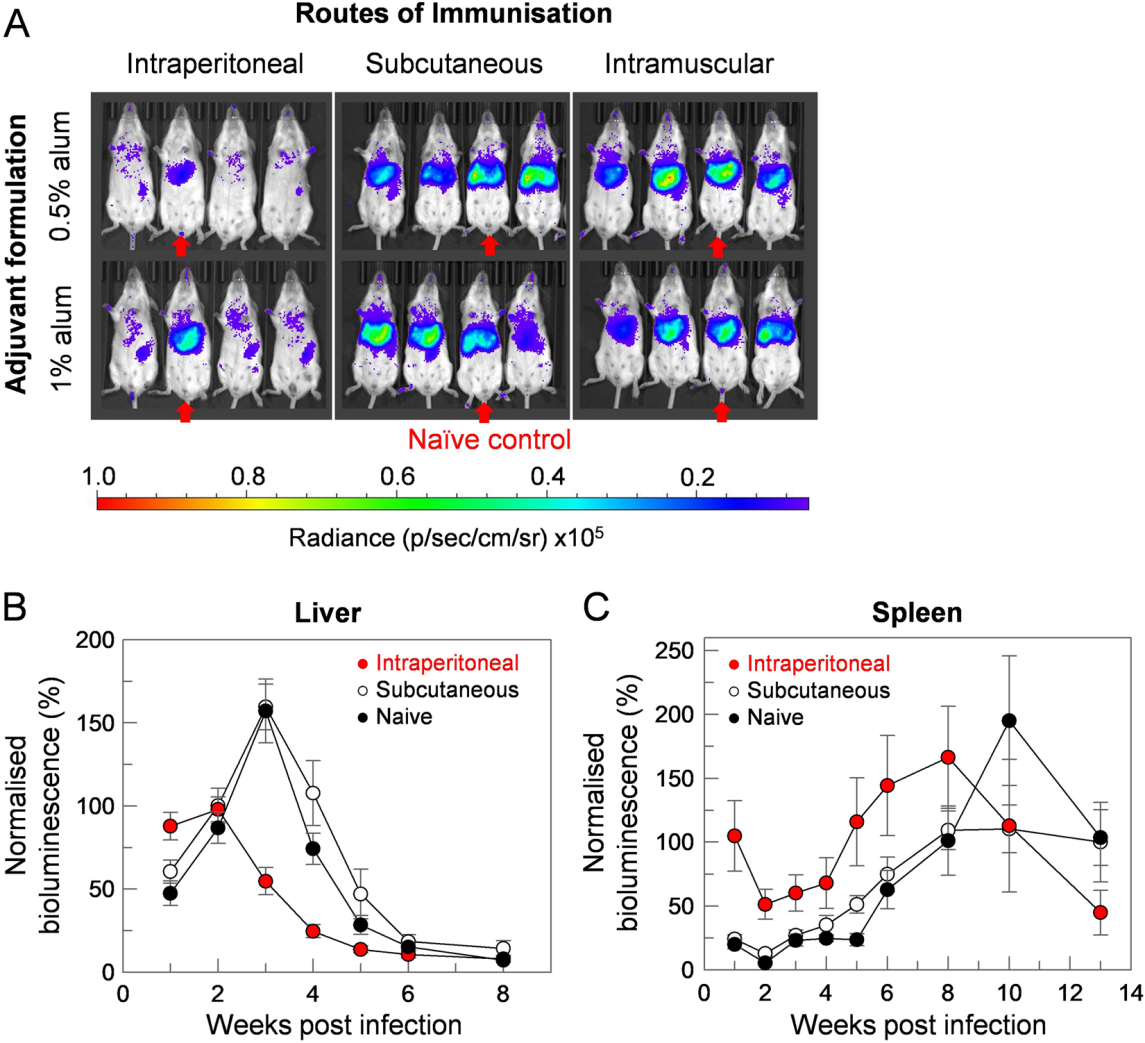
Route of adjuvant formulation delivery affects disease pathology in a murine model of *L. donovani* infection. (**A**) Groups of five mice were immunised via intraperitoneal, subcutaneous or intramuscular routes of injection with a control protein expressed in a mammalian expression system adsorbed to 1% or 0.5% alum. Mice were challenged intravenously with 10^8^ stationary-phase parasites, and an unimmunised mouse is present within each group (identified by red arrows) as a control. Infections were monitored by IVIS after 1 week and those immunised in the peritoneal cavity showed a reduction in parasite burden compared to the naïve control mice and those immunised by subcutaneous and intramuscular routes. (**B,C**) Groups of five mice were immunised via intraperitoneal or subcutaneous routes of injection with control protein adsorbed to 1% alum, challenged intravenously with 10^8^ stationary-phase parasites and infections monitored compared to unimmunised naïve mice by *in vivo* imaging for 13 weeks. Bioluminescence (total flux, photon/sec) was quantified around defined regions of interest overlying the liver (**B**) and spleen (**C**) for naïve (black circles), subcutaneously-immunised (white circles) or intraperitoneally-immunised (red circles) animals. Data points represent means ± S.E.M. (*n* = 5). Relative bioluminescence was calculated by arbitrary normalisation to signals at week 2 (liver) and week 12 (spleen) of subcutaneously-immunised animals.

The protective outcome of a protein-in-adjuvant formulation can sometimes be dependent on its route of administration and some adjuvants may cause general immunostimulatory effects that could affect the infection parameters of a disease model. To determine a vaccine formulation that would be compatible with our infection model, groups of mice were vaccinated with a control protein expressed using a mammalian expression system that we have shown is suitable for expressing extracellular parasite proteins in a biochemically active form^44^. We selected alum as a mild, human-compatible adjuvant and quantified the initial progression of the infection in the liver when animals were vaccinated by one of three different routes: intraperitoneal, intramuscular or subcutaneous (Fig. 4). To reduce any general immunostimulatory effect of vaccine administration, mice were rested for four weeks after the final immunisation prior to an infection challenge. Mice were immunised with a control protein rat Cd200_Cd4^44^ enabling us to quantify the elicited antibody response and compare different immunisation regimens. While we have repeatedly observed that antigen administration in the peritoneal cavity can elicit higher antibody titres compared to the subcutaneous and intramuscular routes (Fig. S3), surprisingly, control animals vaccinated through this route elicited partial protection against subsequent *L. donovani* liver parasitisation (Fig. 4A). Although antibody titres were lower with intramuscular and subcutaneous administration routes (Fig. S3), we observed no overt difference in *L. donovani* infection dynamics when compared to unimmunised mice, suggesting these are more suitable administration routes to establish any protective immunological effects elicited by a vaccine (Fig. 4A). To investigate this further, we established that this was likely to be a general immunostimulatory effect of the adjuvant, since mice treated intraperitoneally with alum alone also showed partial protection. To determine if this also affected the subsequent progression of the infection to the spleen, control mice immunised either through the subcutaneous or intraperitoneal routes were followed for 14 weeks. We again observed that mice immunised in the peritoneal cavity, after an initially higher parasitisation of the liver peaking at the second week, resolved the infection much more rapidly compared to unimmunised mice or those immunised subcutaneously (Fig. 4B). Intraperitoneal vaccination promoted parasite localisation to the spleen during the early stages of infections with higher splenic parasite loads consistently measured in the first eight weeks, and this resolved faster after week eight compared to unimmunised naïve and subcutaneously vaccinated mice (Fig. 4C). Because we observed little difference in infection progression between naïve mice and animals immunized subcutaneously, all vaccine formulations were subsequently administered subcutaneously.

### Mice that resolved initial infection are resistant to reinfection

**Figure 5:**
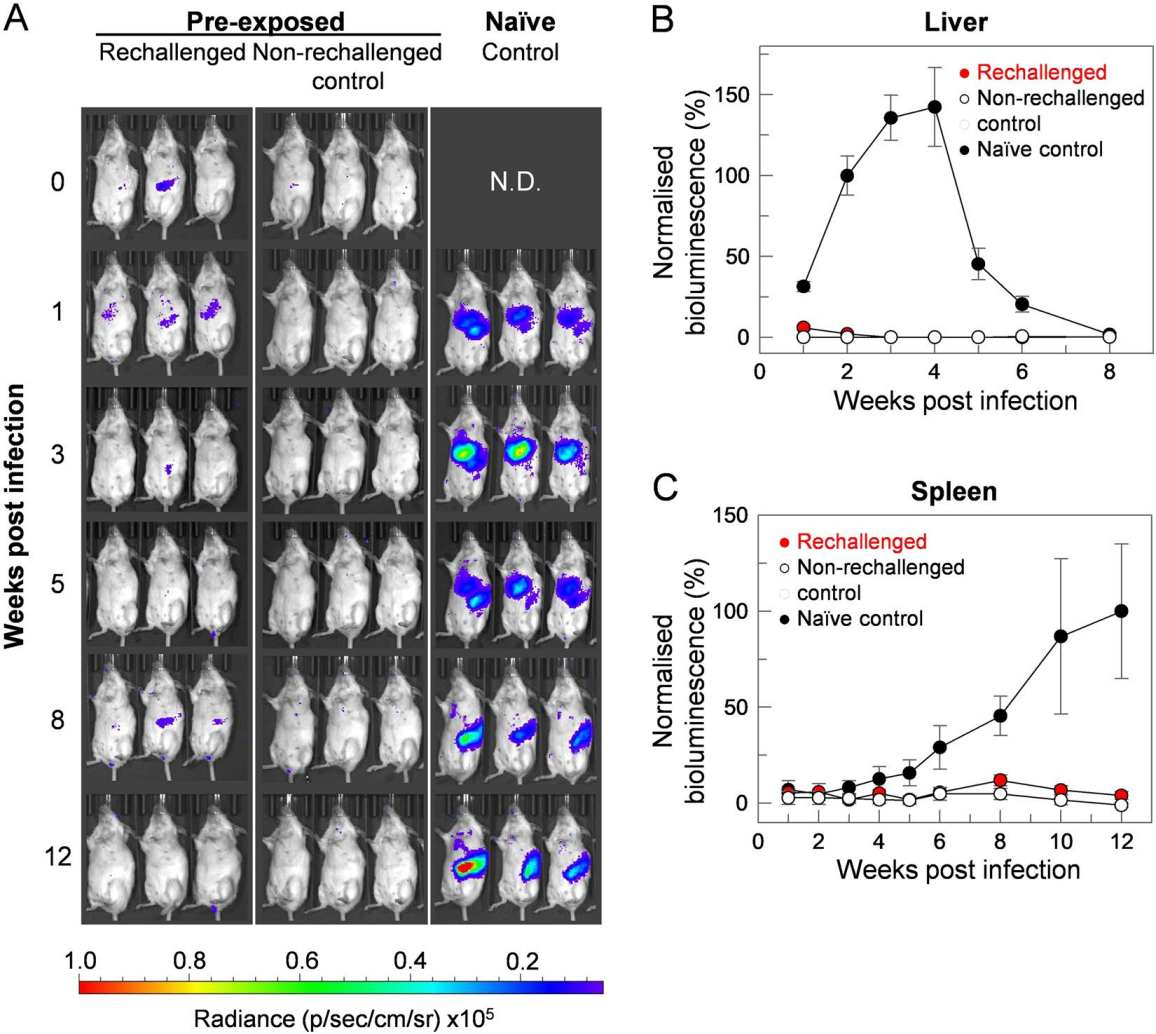
Mice that resolved an initial infection are resistant to reinfection with 10^8^ parasites. (**A**) Two groups of five mice were pre-exposed to live parasites by intravenously infecting them with a suboptimal dose of 10^7^ parasites per mouse; after 16 weeks, no parasites were detected by *in vivo* imaging. One group was then reinfected intravenously with 10^8^ parasites (week 0), while the second group served as a control for any possible disease progression from the pre-exposure infection. A third group of naïve control mice was infected intravenously with 10^8^ parasites as a positive control for virulence of the inoculum. By contrast to controls, mice that had been previously exposed to live parasites were immune to subsequent reinfection. Three representative animals from the group are shown and are arranged so that the infection dynamics can be followed in each individual animal throughout the course of the infection. (**B**,**C**) Bioluminesence (total flux, photon/sec) were quantified around defined regions of interest corresponding to the liver (**B**) and spleen (**C**). Group 1 (red circles, *n* = 4), Group 2 (white circles, *n* = 5) or Group 3 (black circles, *n* = 9) animals. Data points represent means ± S.E.M. of measurements. Relative bioluminescence was calculated by arbitrary normalisation to signals at week 2 (liver) and week 12 (spleen) of naive control-infected animals.

The 1 × 10^8^ dose of parasites required to achieve repeatable experimental infections for comparative vaccine screening led to a concern that immunity could be overwhelmed and thereby mask any vaccine-elicited protective effects in our model. It is known that mice which have resolved an initial VL infection can generate protective immune responses making them resistant to subsequent infections^45^. To demonstrate that our infection model could be used to identify protective effects, we attempted to induce immunity by infecting mice with a dose of live parasites that could be cleared in a few weeks. Consistent with earlier experiments (Fig. 3A), mice infected with a dose of 10^7^ parasites had reduced parasite loads in both their livers and spleens which gradually resolved so that in some animals it fell below the limit of detection by week 12 (Fig. S4). To determine if immunity was elicited, these mice were subsequently infected with 10^8^ parasites which resulted in robust infections in non-immune control mice. We observed that those mice which had previously been exposed to the parasite were protected from subsequent *L. donovani* challenge compared to controls (Fig. 5). This protection included an inability to colonise the liver (Fig. 5B) and spleen (Fig. 5C). Together these data demonstrate that immunity to a challenge of 10^8^ parasites in the infection model could be elicited by prior exposure to a lower dose of parasites.

## Discussion

With the aim of establishing a reproducible and quantitative experimental infection model of VL for systematic vaccine testing, we have selected a transgenic strain of *L. donovani* constitutively expressing an mCherry-luciferase fusion protein and characterised the infection parameters in mice. This model allowed us to visually and quantitatively monitor parasite loads of infected visceral organs in individual mice by *in vivo* imaging and thereby establish important parameters such as the dose of parasite and route of infection.

One major advantage of this model which we believe will make it particularly suitable for systematic vaccine screening is that infections can be longitudinally monitored in individual mice. This helps to account for the heterogeneity observed in individual infections, and the method considerably reduces the numbers of animals that would be otherwise required if parasite loads were quantified using terminal procedures such as classical tissue impression smears or limiting dilution methods. Indeed, we have found *in vivo* imaging with the transgenic parasites to be more sensitive, less subjective and time-consuming than these more established methods of quantifying infections. There are additional challenges, however, that would further improve the model such as more sophisticated higher resolution three-dimensional imaging that would more precisely localise the parasites within infected tissues.

Previous reports of experimentally infecting mice with visceralising *Leishmania* species have exclusively used intravenous delivery, without other routes being explicitly described^46–48^. We have investigated and demonstrated how the parasites persisted at sites of injection with non-intravenous routes before being resolved. A possible explanation for differences in pathology could be the development of protective versus suppressive adaptive immune responses induced by different inoculation routes and parasite doses as suggested previously^23^. Other possible reasons could be the availability of specific host immune components required for interaction with parasite surface molecules or to facilitate subversion mechanisms for macrophage infection establishment^49^. Promastigote metacyclogenesis is an important virulence determinant for *Leishmania* spp. infections^50,51^, since metacyclic parasites are more resistant to serum complement lysis^52–54^. Axenic cultured parasites differentiate to metacyclic promastigotes during stationary phase with nutrient depletion being reported as promoting factors^55,56^. Our study indicated only a subfraction of parasites (~3%) persisted in the livers of infected mice 24 hours following an infection challenge. Interestingly, Ficoll gradient centrifugation^43^ can be used to enrich for metacyclic promastigotes, and in our hands, typically yield 1–3% recovery. It is entirely possible that the persistent ~3% of bioluminescence signal in the liver correspond to the parasites that have differentiated into metacyclogenic forms and able to resist responses mounted by the host immune system. We were, however, unable to establish a robust measurable murine infection using the small number (10^6^) of Ficoll-gradient enriched cells, suggesting other factors such as presence of dead or moribund parasites in the innoculum may also be required^57^.

This infection model does not attempt to replicate the dose and route of natural parasite infection by sand fly bite but rather establish an experimental model that could be used to assess candidates in a comparative and quantitative manner. By inoculating a precisely quantified dose of parasites directly into the circulation, we can reduce some variables such as differences in the number of transmitted parasites during a sand fly blood meal. A similar approach has been very useful to reduce variability and increase the sensitivity to assess vaccine efficacy in controlled human malaria infections by delivering a precise number of cryopreserved *P. falciparum* sporozoites by needle rather than through mosquito bite^58^. Our finding that the parasite growth in the liver in the first few weeks of infection was linear and correlated with the dose of parasites provides a sensitive system to assess even partial effects of vaccines. Importantly, we have established that immunity can be established in our murine infection model by demonstrating the resistance of mice to reinfection following successful resolution of a prior infection, in good agreement with previous studies^45,59^. It also gave us further confidence that the dose of 10^8^ parasites which resulted in repeatable and robust infections does not overwhelm the capacity of the animals’ immune system to resist infections after immunity was acquired.

In vaccine development, adjuvants are usually required to elicit appropriate and long-lasting protective immune responses, particularly with subunit proteins that often lack intrinsic immunogenicity. The immunological mechanisms of action of some adjuvants are not yet fully understood, but are likely to involve the stimulation of multiple components of the host immune system^60^. Although many adjuvants have been developed and optimised to stimulate specific immune responses, only a handful have been approved for use in humans. Since the most common human-compatible adjuvant used in vaccines are aluminium salts (alum) which are used in the majority of licensed human vaccines, its suitability for use against VL was investigated in our model. More importantly, soluble leishmania surface antigens adjuvanted with alum were previously shown to be protective in a murine VL infection model^61^. To minimise and account for general adjuvant-based stimulation of host immunity, we have incorporated a “resting” period into our immunisation schedule, where vaccinated animals were “rested” for four weeks following the final boost before being infected. Alum was found to be a suitable adjuvant when administered subcutaneously or intramuscularly but not intraperitoneally. Although intraperitoneal administration would not be considered for humans, these findings highlight important considerations when using alum or any other adjuvants in vaccinology studies against VL or other diseases. In summary, we have established a robust, sensitive, and reproducible murine experimental infection model of visceral leishmaniasis. We envisage that this model will provide an important platform to facilitate systematic testing of the many subunit vaccine candidates that have been identified from the recent advances in genome sequencing and gene annotation. The eventual goal will be to use this model to identify a range of potential vaccine targets to help treat and prevent this deadly disease.

## Methods

### Ethics statement

All animal experiments were performed under UK Home Office governmental regulations and European directive 2010/63/EU. Research was approved by the Sanger Institute Animal Welfare and Ethical Review Board.

### Generation of transgenic *L. donovani* expressing mCherry and Luciferase

Transgenic parasites were generated by transfection of the wild-type LV9 strain of *L. donovani* with an integrating construct leading to stable luciferase expression. Briefly, the gene firefly luciferase was cloned into the XmaI site (SmaI isoschizomer) of pIR1SAT, an integrating vector that was kindly provided to us by Dr. Stephen M. Beverley of Washington University, St. Louis^62^. The vector was digested with SwaI generating products of 2860 and 5630 bp, whose sizes were verified on agarose gel electrophoresis. The reaction product with the two unseparated fragments were introduced by electroporation into promastigotes in logarithmic stage growth by electroporation as described^63^ and selection on semi-solid medium with 100 µg/mL nourseothricin. After 2–4 weeks at 26 °C, drug-resistant clones were picked and amplified in liquid growth medium with 100 ug/mL nourseothricin. Correct homologous integration of the insert was verified by Southern blotting as previously described for the generation of luciferase expressing *L. infantum*^35^ or by PCR of genomic DNA.

### *L. donovani* promastigote culture and animal infections

Parasites were cultured at 28 °C in LdPro medium^64^ supplemented with 100 µg/mL nourseothricin and 10 U/mL penicillin-streptomycin (Gibco). Parasites for infecting animals were prepared by subculturing cells at an initial seeding density of 1 × 10^6^ cells/mL. Stationary phase promastigotes were harvested by centrifugation (2000 × *g*, 10 mins) and washed twice with PBS. Parasites were resuspended at appropriate densities in DMEM medium (Sigma) and used to infect BALB/c mice intravenously, intraperitoneally or subcutaneously at 200 µL/injection/animal. Infections were monitored by *in vivo* imaging at regular defined intervals. To maintain virulence, parasites were harvested from spleens of infected animals as previously described^65^, with minor modifications. Briefly, organs were suspended in LdPro medium (5 mL/g) and homogenised using the gentleMACS Dissociator (Miltenyi Biotec) with M-tubes. Tissue extracts were treated with saponin (50 µg/mL) and clarified by two rounds of centrifugation (200 × *g* and 2000 × *g*) as previously described^65^. Final pellets were resuspended in LdPro medium and incubated at 28 °C to allow amastigotes to differentiate into promastigotes. Aliquots of freshly differentiated promastigotes were stored as stabilates at −80 °C using 10% (v/v) glycerol as a cryoprotectant. To minimise variability between infection challenges, promastigote preparations that had been maintained in culture for no longer than two weeks were routinely used.

### Monitoring infection *in vivo* imaging

D-luciferin (K^+^ salt, BioVision, Inc.) was reconstituted to 30 mg/mL in Dulbecco’s PBS without Ca^2+^ and Mg^2+^ (Hyclone), filter-sterilised (0.22 µm) and stored in aliquots at −20 °C. Thawed aliquots were administered to animals intraperitoneally at 300 mg/kg. Injected animals were allowed free movement for three minutes before being anaesthetized in an anaesthesia chamber attached to the *In Vivo* Imaging System (IVIS Spectrum, PerkinElmer) using 3% gaseous isoflurane. Anaesthetised mice were moved to the imaging chamber and general anaesthesia was maintained using 3% gaseous isoflurane administered through built-in nose cones attached to a manifold until data acquisition was completed. Bioluminescence acquisitions were performed ten minutes after luciferin injection using fixed parameters of exposure 3 min, binning medium 8.0 and f/stop 1.0, respectively. Data were analysed using the Living Image software, with bioluminescence signal converted to radiance (photons/sec/cm^2^/sr) using a fixed normalised scale on IVIS images of animals. Bioluminescence quantified around defined regions of interest overlying to the location of the liver or spleen was expressed as total flux (photons/sec) and analysed using GraFit. IVIS images were normalised to a fixed scale with radiance limits set at 5 × 10^3^ (minimum) and 1 × 10^5^ (maximum).

### Immunisations and determination of antibody titres

Mice used in the study were produced by the Research Support Facility at the Wellcome Sanger Institute. Rat Cd200-Cd4 fusion protein was expressed in a mammalian expression system (HEK293 cells) and purified as previously described^44^ and used as a control protein for immunisations to quantify immune responses. Control proteins were mixed with 2% alum (Alhydrogel, InvivoGen) at a 1:1 (v/v) ratio and incubated on a roller mixer for 1 h at 22 °C. Groups of BALB/c mice (6–7 weeks old) were immunised by injecting the protein-alum mix either intraperitoneally (200 µL into peritoneal cavity), subcutaneously (2 × 100 µL into flank regions) or intramuscularly (2 × 50 µL into muscles in the hind legs). Booster shots were similarly prepared and administered on days 14 and 28 following the initial immunisation. Control proteins were administered to mice at 100 µg and 20 µg per animal in the initial immunisation and subsequent boosters, respectively. Sera were harvested from tail bleeds of immunised mice 8 days after the final immunisation, with antibody titres determined using ELISA as previously described^66^. Briefly, a fixed concentration of the biotinylated control protein was immobilised on 96-well streptavidin plates (Thermo Scientific) and incubated with serial dilutions of the corresponding sera prepared in HBS/0.1% (v/v) Tween 20 (HBST), containing 2% (w/v) BSA. This was followed by three washes with HBST before incubation with a secondary alkaline phosphatase (AP) conjugated anti-mouse IgG (Sigma) for 1 h. Following three further washes with HBST, AP substrate (100 µL, 1 mg/mL in ELISA buffer, Sigma) was added and AP activity was quantified at 405 nm using a plate reader (Tecan). Results were analysed using four parameter non-linear regression in GraFit. Mice were infected with *L. donovani* intravenously 28 days after the final immunisation.

## Supplementary information

Supplementary Information.

## Data Availability

All data are available in the article text and figures or supplementary files, and available from the corresponding author upon reasonable request.
