# Author Correction: Establishment, optimisation and quantitation of a bioluminescent murine infection model of visceral leishmaniasis for systematic vaccine screening

**DOI:** 10.1038/s41598-021-87190-2

**Published:** 2021-04-07

**Authors:** Han Boon Ong, Simon Clare, Adam Jonathan Roberts, Mary Edythe Wilson, Gavin James Wright

**Affiliations:** 1 Cell Surface Signalling Laboratory, Wellcome Sanger Institute, Cambridge, UK; 2 Pathogen Laboratory Support, Wellcome Sanger Institute, Cambridge, UK; 3 Departments of Microbiology and Immunology and Internal Medicine, University of Iowa, Iowa City Veterans’ Affairs Medical Center, Iowa City, USA

Correction to: *Scientific Reports* https://doi.org/10.1038/s41598-020-61662-3, published online 13 March 2020

The original version of this Article contained a typographical error in the spelling of the author Mary Edythe Wilson, which was incorrectly given as Mary Elizabeth Wilson. This has now been corrected in the PDF and HTML versions of the Article, and in the accompanying Supplementary Information file.

