# Supplementary material for: Establishment, optimisation and quantitation of a bioluminescent murine infection model of visceral leishmaniasis for systematic vaccine screening
Source: Sci Rep. 2020 Mar 13;10:4689. doi: 10.1038/s41598-020-61662-3 (PMC7070049; doi:10.1038/s41598-020-61662-3)
Supplement: Supplementary file 1 — Supplementary Information. [file 41598_2020_61662_MOESM1_ESM.pdf]

Supplementary Information

Establishment, optimisation and quantitation of a bioluminescent murine infection model of visceral leishmaniasis for systematic vaccine screening

Han Boon Ong[1], Simon Clare[2], Adam Jonathan Roberts[1], Mary Edythe Wilson[3] and Gavin James Wright[1,*]

[1] Cell Surface Signalling Laboratory, Wellcome Sanger Institute, Cambridge, UK.

[2] Pathogen Laboratory Support, Wellcome Sanger Institute, Cambridge, UK.

[3] Departments of Microbiology and Immunology and Internal Medicine, University of Iowa, and the Iowa City Veterans' Affairs Medical Center, Iowa City, USA

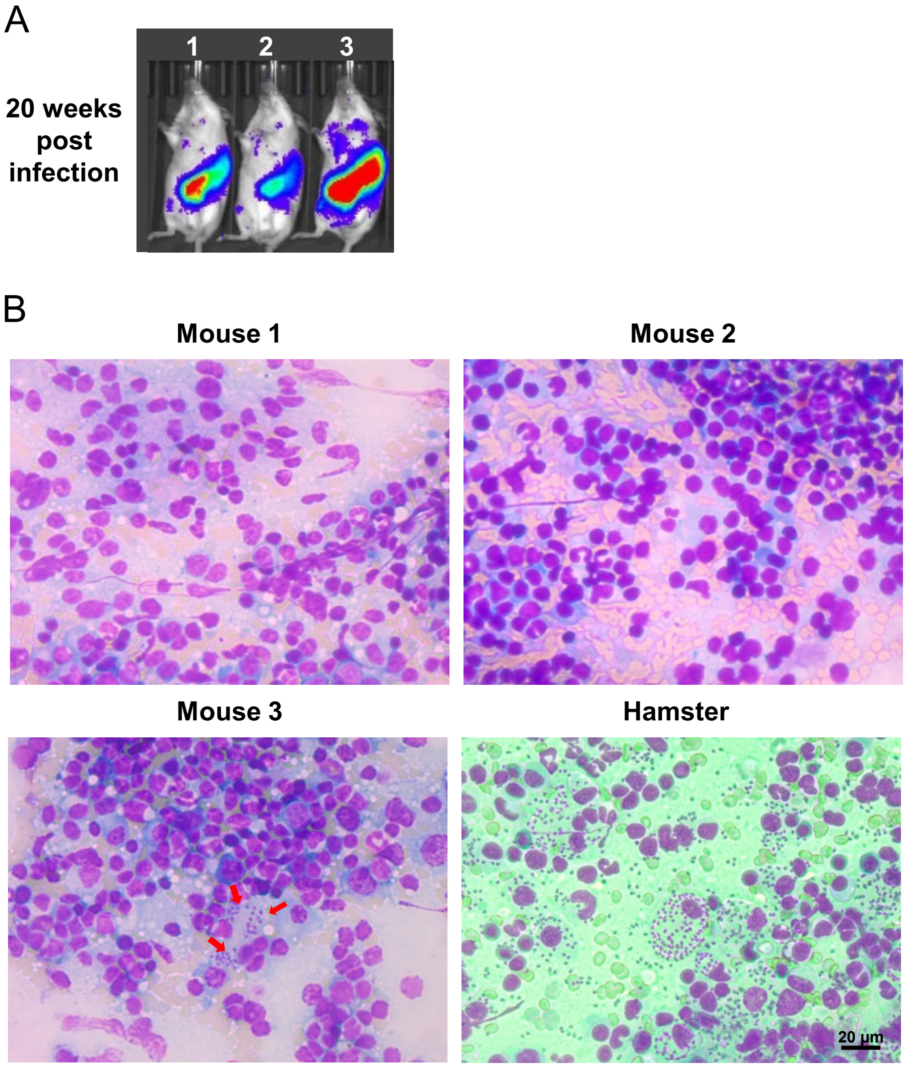

Figure S1 Parasite-infected cells can be observed in highly bioluminescent animals. (**A**) Groups of three mice were infected intravenously with 0.5 x 10^8 parasites and animals were imaged and then euthanised at 20 weeks post infection and spleens harvested for quantitative analyses. (**B**) Impression smears were prepared from the infected mouse spleens with differing bioluminescence signals. Of the prepared smears, infected cells (identified by red arrows) were only observed in one out of fifty fields of view from mouse 3 which exhibited the highest bioluminescence signal. No parasites were observed in the smears prepared from mice 1 and 2 which had lower associated bioluminescence suggesting that the bioluminescence is more sensitive at detecting parasites than impression smears. As a control, impression smears were prepared from a wild-type L. donovani-infected hamster spleen which had lost 10% of its mass following infection; here, heavily infected cells can be observed in every field of view. Smears were stained with RapiDiff staining set as per manufacturer's instructions.

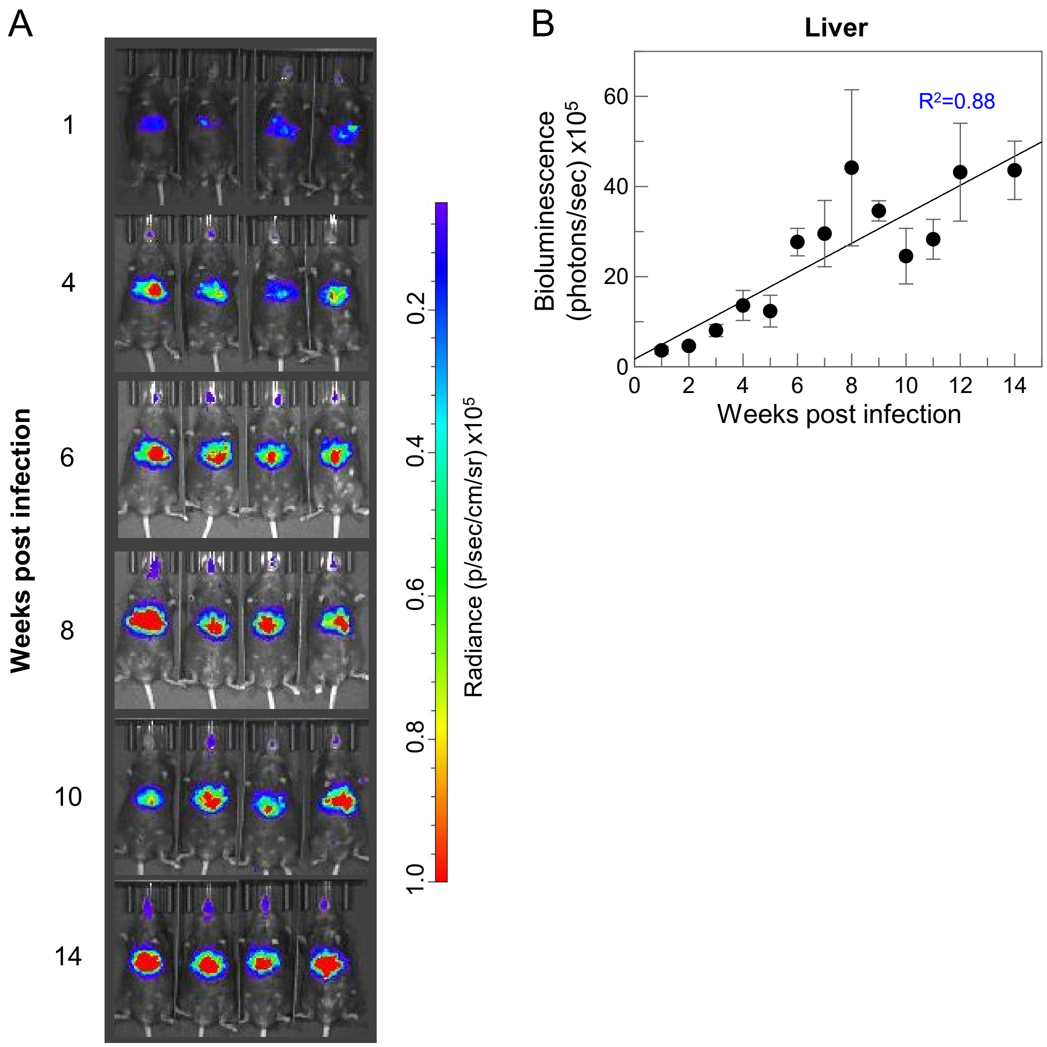

Figure S2 Progression of L. donovani infections in Rag1-deficient immunocompromised mice. (**A**) Four Rag1-deficient mice were infected with 10^8 day 7 stationary-phase L. donovani promastigotes intravenously. Infections were monitored over 14 weeks by in vivo imaging. (**B**) Hepatic parasite load was quantified by measuring the liver bioluminesence (total flux, photon/sec) and plotted as a function of time. Data points represent means ± SEM; (*n* = 4) and were analysed using linear regression in GraFit (R^2 = 0.88).

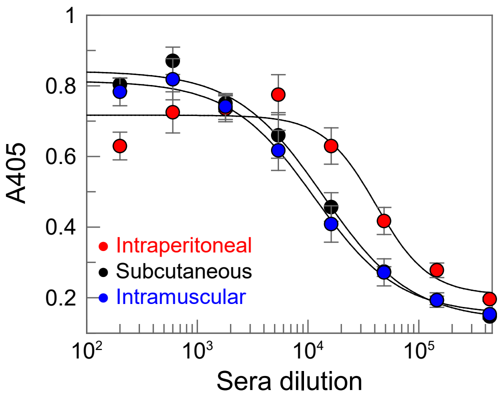

Half maximal IgG titre

Route	50% alum	25% alum
Intramuscular	● 11000 ± 1300	3900 ± 780
Subcutaneous	● 14000 ± 2500	8500 ± 860
Intraperitoneal	● 41000 ± 12000	25000 ± 6700

Figure S3 Half maximal sera IgG titres curves of sera collected from mice immunised with control protein via different routes. Sera were prepared from tail bleeds of mice immunised with the control protein delivered intraperitoneally, subcutaneously or intramuscularly. Sera were serially diluted and incubated with a fixed concentration of antigen followed by anti-mouse alkaline phosphatase-conjugated secondary antibody. Antibody titres were quantified by measuring the hydrolysis of a colorimetric phosphatase substrate at 405 nm using a plate reader and calculating the sera dilution required for the half-maximal response. Intraperitoneal administration resulted in ~3-fold higher IgG antibody titres than other delivery routes. Data points represent means ± S.E.M of single measurements from four individual animals.

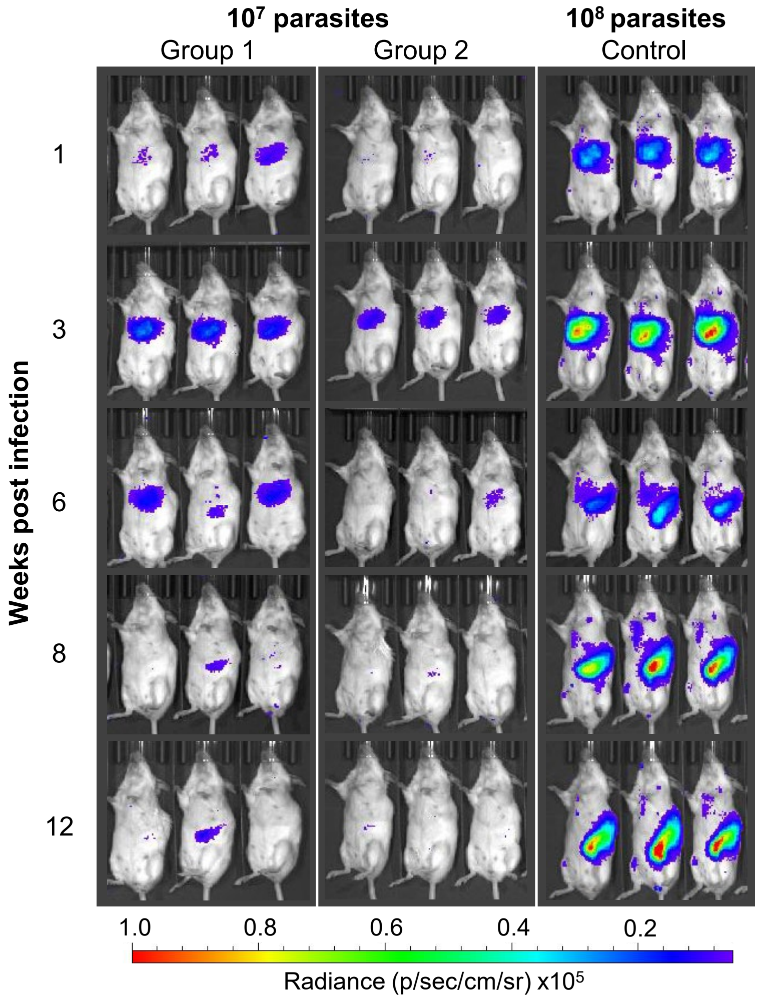

Figure S4 Disease progression and resolution of mice pre-exposed to 10^7 parasites to induce immunity. With the aim of pre-exposing mice to induce immunity, two groups of five mice (Groups 1 and 2) were immunised and infected intravenously with 10^7 parasites while the control group (Group 3) was given 10^8 parasites to confirm the virulence of the parasite preparation; infections were monitored by *in vivo* imaging over 12 weeks. By contrast to animals receiving 10^8 parasites which all developed robust infections as expected, those mice receiving 10^7 parasites exhibited lower liver parasite loads which resolved by week twelve. These animals were subsequently used in challenges to determine if immunity had been elicited. Three representative animals from the group are shown and are arranged so that the infection dynamics can be followed in each individual animal throughout the course of the infection.
